# Classification of ransomware using different types of neural networks

**DOI:** 10.1038/s41598-022-08504-6

**Published:** 2022-03-19

**Authors:** Houria Madani, Noura Ouerdi, Ahmed Boumesaoud, Abdelmalek Azizi

**Affiliations:** Faculty of Sciences, Mohammed First University, Oujda, Morocco

**Keywords:** Computer science, Information technology

## Abstract

Malware threat the security of computers and Internet. Among the diversity of malware, we have “ransomware”. Its main objective is to prevent and block access to user data and computers in exchange for a ransom, once paid, the data will be liberated. Researchers and developers are rushing to find reliable and safe techniques and methods to detect Ransomware to protect the Internet user from such threats. Among the techniques generally used to detect malware are machine learning techniques. In this paper, we will discuss the different types of neural networks, the related work of each type, aiming at the classification of malware in general and ransomware in particular. After this study, we will talk about the adopted methodology for the implementation of our neural network model (multilayer perceptron). We tested this model, firstly, with the binary detection whether it is malware or goodware, and secondly, with the classification of the nine families of Ransomware by taking the vector of our previous work and we will make a comparison of the accuracy rate of the instances that are correctly classified.

## Introduction

The growth of malware attacks is affecting IT teams and any Internet user’s. A lot of research is being conducted to find a solution to this problem. Malware can undoubtedly sneak into our PCs without knowing how or when we are affected. Each type of malware has a degree of danger, there are some that hide and do the job without the client realizing it, others that crash the PC when executed, as well as others that take the individual information of the designated client.

We take one of the dangerous malwares, the “Ransomware”. It comes from the name “Ransom-Malware”, it is a malware which targets clients to steal their information, encrypt their files and prevent them from accessing their computer under the compulsion of paying a ransom to send them the decryption key in order to free the data. This type of infection, in addition to its danger, it is lucrative. The issue of payment is discussed by researchers and specialists in the security field^1,2^, because if the victim pays, it opens an opportunity for attackers to threaten and attack him again.

This paper presents the continuation of the work presented in the conference paper^3^ with an improvement of the work using two others types of neural networks. For example, if we take a file (executable), in the first step, we detect whether it is malware (ransomware) or goodware. In the second step, if the detected file is a ransomware, we can identify the class of this ransomware.

In the “Related works” section will discuss the related works to get an idea about similar works and to be update with the new methods used. In the “Methodology” section, we will detail our methodology. The part of the results and discussion will be treated in the “Results and discussion” section. At the end, we will close our paper with a general conclusion.

## Related works

Recently, malware classification and detection have become very competitive among researchers^4^, each one uses a method to prove the effectiveness of its results.

We often see the use of machine learning techniques with their different algorithms and especially neural networks because they are able to analyze in depth the ransomware behavior^5^. Among the proposed ransomware classification methods, the authors^6^ suggested an approach using machine learning algorithms which have been used for binary classification of ransomware using static analysis of opcodes transformed into n-gram, they achieved an accuracy of 91.43%.

### Classification of malware using artificial neural networks (ANN)

Rad et al.^7^ have collected different types of malware from a malicious repository shared by Naiyarah Hussain^8^. The authors used as many samples as possible (both benign and malicious), the samples are placed as follows:

Malicious samples are placed in the “malicious_samples” folder and the benign samples are placed in the “benign_samples” folder. With this way, they can dispense with manually labeling each obtained file. To ensure that the samples obtained are usable and properly labelled (malicious or benign), they proposed criteria the following criteria:

- An executable file (PE for Portable Executable) is valid by checking the first 2 bytes of the binary file if it contains “MZ”.
- Exclude packaged malware from the dataset so as not to damage the classification model by verifying the signature of each piece of software to see if it is packaged by a packer (such as UPX) or not.
- The collected samples are compared with anti-viruses. If the malicious sample is detected (even at a low rate), it will be removed. For the benign sample, if the anti-virus has not detected anything (with a rate of 0%), the sample will be in the list of benign samples.

The authors required the relevance and effectiveness of the aforementioned criteria in order to remove any sample that might affect the model that they want to realize. They proposed a neural network which does a binary classification of an invisible PE (Portable Executable) file as a begnin or malicious, developed with the Python library « TensorFlow-GPU». In addition, they used «scikit-learn» to do the cross-validation method. The dataset contained 4000 samples (1000 benign and 3000 malicious), each class is represented by vector indicating (0, 1) if the sample is malicious and (1, 0) if it is benign. The input size (Windows library function calls) was 34087 after the pre-processing of the dataset. They validated the model using the cross-validation method, they have assumed that the model can perform on an unknown dataset, and will operate based on the results that have been achieved. With this model, they obtained an average accuracy of 97.8%, with 97.6% of precision and 96.6% of recall.

Malware comes in several types; our goal is to focus on ransomware. The paper^9^ presents our previous work, which was a comparative study of a proposed ransomware dataset on work^10^ with a new proposed dataset. We changed the learning files but we did not have a test dataset, so we took 20% of the learning database file to build our test database. The results were high compared to those in paper^10^ but the problem was that the 20% of files taken, was not removed from the learning database, we copied the test part in question to another file. We obtained approximately a rate of 70% for correctly classified instances, but they were not as surprising and relevant as expected. We also compared the two algorithms **A**rtificial **N**eural **N**etwork (ANN) and **B**ayesian **N**etwork (BN), to deduce the one that gives us the best results in our work, and we got the right results with the ANN.

### Classification of malware using convolutional neural networks (CNN)

Many researchers use CNN to classify and detect malware. Kabanga et al.^11^ proposed a model of convolutional neural networks to extract features from images at a time, these images extracted from a sample of malware will be classified. The model has images of a size 128*128*1 (1 being the channel width), they used the image library from the PIL (Python Image Library) package of Python to generate vectors of images and after the generation of the vectors, processing was performed on these vectors. Then, they designed a three-layer deep convolutional neural network to do the classification task. The output layer has 25 neurons that correspond to the 25 families of malware available in the dataset used. The goal of this neural network is to have a single class containing the malware. The Malimg dataset^12^ that was used, has 25 malware families (with several grayscale images), which 90% are used for training and 10% for testing. They obtained in the results an accuracy of 98%. Using this technique, they have proven that it is the best compared to other traditional methods of classification. However, using images to classify malware can lead to erroneous results due to poor image extraction.

### Classification of malware using recurrent neural networks (RNN)

Many researchers have used RNN to do speech recognition and automatic natural language processing, and they have had pertinent results, the question we ask is as follows: can we use recurrent neural networks to classify and/or detect malware?

Pascanu et al.^13^ attempt to learn the language of malware in order to detect it by constructing a recurrent model to predict the next API call using the hidden state of the model (which encodes the history of past events) as a fixed-length vector that is given to a classifier (by the logistic regression or Multi-layer Perceptron “MLP” algorithm). And to improve their recurrent model, the authors introduce Max-Pooling on the values of the hidden units in the input sequence, and they propose the Half-Frame model that increases the memory capacity of the final representation by including the mid-state information of the sequence of events in the final state. The authors suppose that the memory window of the standard ESN (Echo State Network) and RNN models is not adequate, to solve that, a Leaky-Units architecture^14^ has been added to the recurrent architectures, which allows the increase of the system memory in the long term.

In paper^15^, The authors added a bidirectional mode that combines two distinct models, one learning by treating events in the forward direction and the other learning by processing the events in the reverse direction.

#### Fixed-length representations

After the weights have been learned for the RNN or generated for the ESN, the next processing step is to create a fixed-length representation for the event stream provided at the classification input and also to determine the detection time of a malicious or non-malicious file. In order to do this, they decided to eliminate sequences shorter than 15 and explore the values 50, 100, 200, and 65,536 of sequences longer than N steps, which is considered a hyper-parameter. Only the first N events was selected.

#### Classification

They used both logistic regression and multi-layer perceptrons with rectifier units^16^ to classify fixed-length projections and also Dropouts which showed a significant improvement in the generalization of the MLP model^17^.

RNN and ESN are formed independently from the classifier. Thus, they act as feature extractors trained in an unsupervised manner. The results show that the combination of a recurrent model with a standard classifier can improve the classification of malware, and on the other hand, ESN models outperform RNN models in the majority of experiments, but the use of recurrent neural networks is a bit complicated. More, we cannot assume that this is the right method to do malware classification and detection because of the need to learn the language of malware, which is a bit difficult considering that malware varies.

Ransomware detection systems can improve their performance by having the ability to capture recurrent (or repetitive) behavior and general sequence learning. That’s why Agrawal et al.^18^ worked on the attention mechanisms while processing executable sequences for ransomware detection. They proposed an implementation of an improved neural cell to integrate the attention mechanism in learning named ARI (Attended Recent Inputs); this ARI is used by LSTM (Long Short-Term Memory) networks, named ARI-LSTM. This cell allows to perform a detailed analysis of ransomware executable, i.e. it processes input sequences by taking the attention weights for each recent input. In the learning phase, they took the LSTM model (improved by incorporating the ARI cell) and Max Pooling (LaMP). The goal of the learning is to classify the input sequences by labeling the ransomware by “1” and the benign by “0”. They used a set of sequence data containing benign and ransomware executable of a Windows operating system captured from user’s computers. The results show that the accuracy of the ARI-LSTM is better than an LSTM, proving the effectiveness of attention mechanisms in the learning phase.

Other than malware detection, the types of neural networks studied in this article have also been successfully applying in other fields, such as:

- Analyzing user’s check-in to predict the locations that they may visit using RNN^19^.
- In order to control greenhouse climate, the authors^20^ propose a model for predicting greenhouse climate by focusing on the climatic factors: crop growth, temperature, humidity, lighting, carbon dioxide concentration, and soil temperature and moisture, using LSTM (Long Short‐Term Memory) to capture the reliance between historical climate data.
- Combining the attention mechanism with bidirectional gated recurrent units (GRU) to increase the prediction of the point of interest (POI) category, in order to mitigate the scarcity of users’ check-in data during the Coronavirus (COVID-19) period^21^.

## Methodology

### Objective

The objective of this work is to study, in first step, if we can detect a program as goodware or malware according to the system calls it provides, and, in the second step, we will see malware classification (precisely ransomware families) based on the previous work^3^ by applying the implemented model in different types of neural network and we are going to compare the result in work^9^ with the new one.

### Binary classification (goodware or malware)

#### Implementation of the multilayer perceptron (MLP)

**Figure 1:**
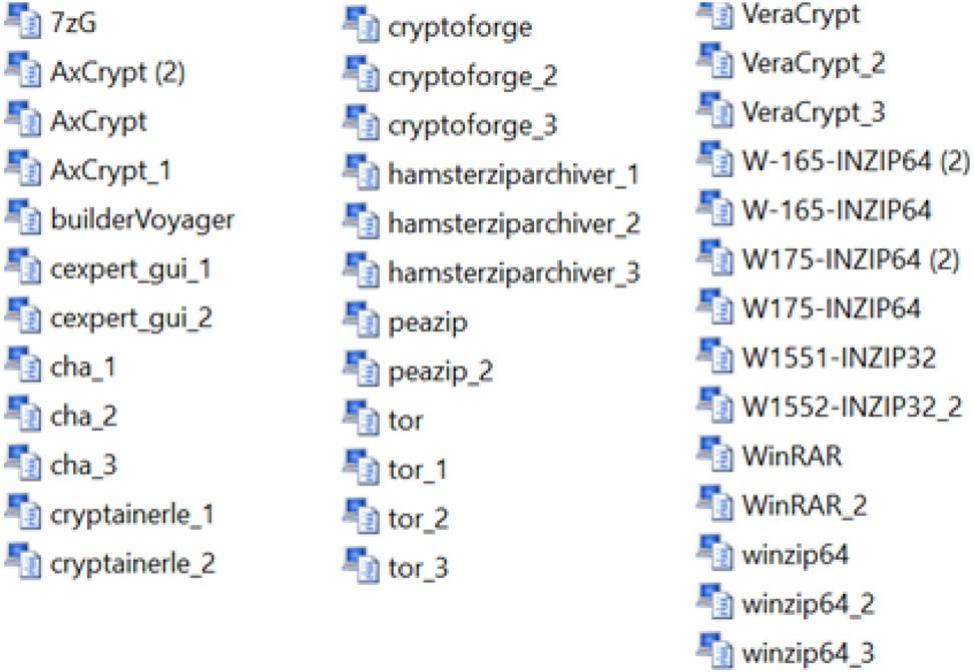
Goodware dataset.

We took a data set that contains 38 dump files representing goodware files (Fig. 1). Dump files (also called backup files) are automatically generated by Windows when a program stops running or when the computer crashes, these files are used to discover the error that caused the dysfunctioning.

**Figure 2:**
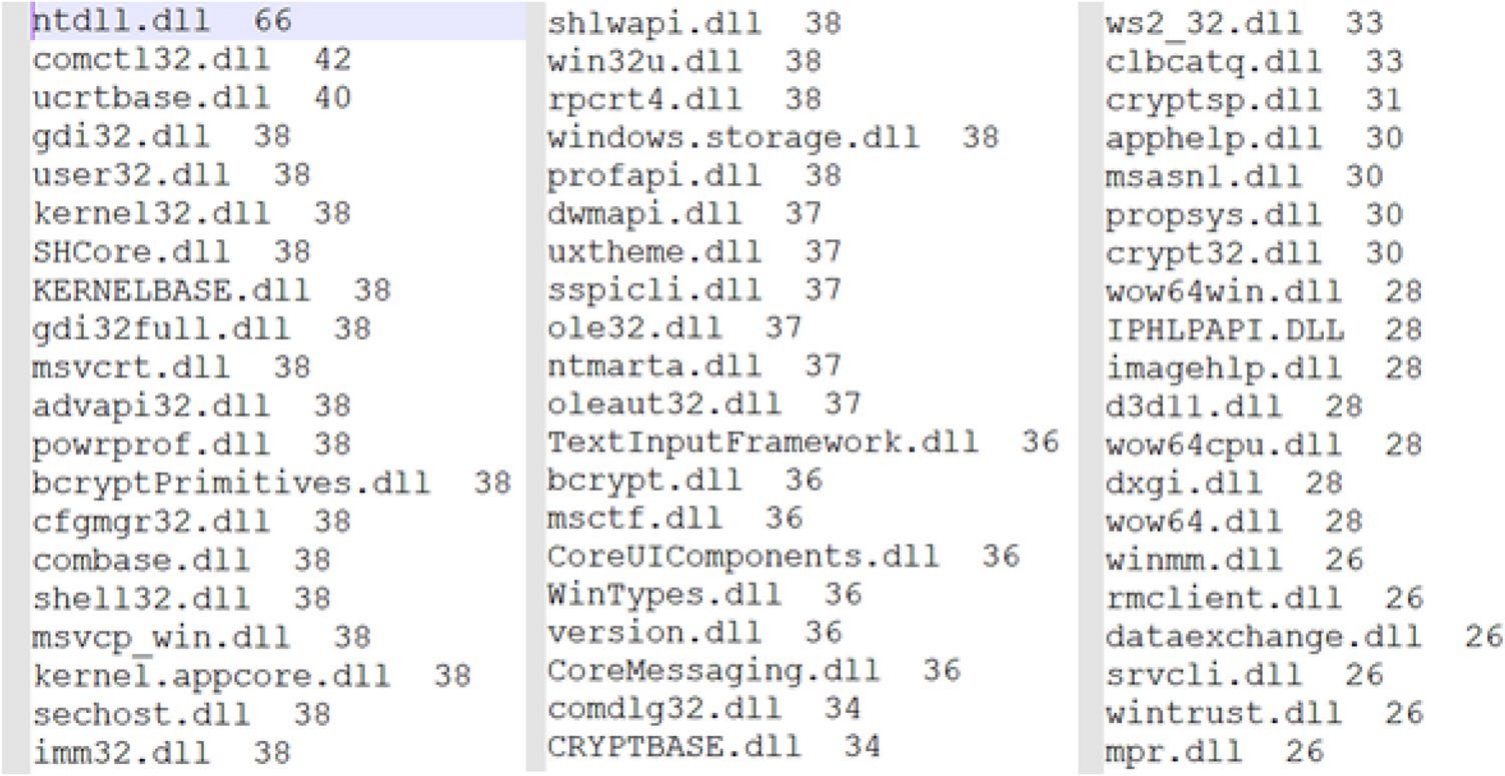
The first 60 strings of the vector.

1. *Collection of system calls (DLL)* We browsed through all the 38 dump files, and gathered their contents into a single file, resulting in 4612 strings (system calls).
2. *Search for distinct Strings* Among the 4612 strings, we extracted the distinct strings, in order to build a single V vector to instantiate the different goodware by developing a Java program. We found 445 distinct strings, so the length of the vector is equal to 445. (Fig. 2) shows the first 60 strings with the number of occurrences sorted in descending order.
3. *Instantiation of vector V* Once the V vector is constructed, we developed a Java program to instantiate the goodware of the dataset. If the String exists in the goodware, we put “1”, else we put “0”. We obtained 38 instances of the vector V (38 instances correspond to the number of files). Each instance of value 0 or 1 represents the associated goodware.
4. *The learning base and the test base* To build the learning base and the test base, we have 38 instances of vector V as the database; we took 30 instances for learning and 8 instances for testing.
5. *The structure of the neural network model* The type of neural network we have chosen to develop is a multilayer perceptron. The model is developed using Java language, with 445 neurons in the input layer, 445 neurons in the hidden layer, and a single neuron in the output layer to solve the binary classification problem (1 corresponds to “goodware” and 0 corresponds to “malware”).
6. *Learning Algorithm* In order to do the learning, the following steps were followed:
  - Initialization of the weights ${\text{w}}_{{\text{hj }}}$ randomly for the input layer.
  - Initialization of the inputs ${\text{x}}_{{\text{j }}}$ for the input layer.
  - Propagation from the input layer to the hidden layer using the following formula (1):
    1 $$ z_{h } = f\left( {\mathop \sum \limits_{j = 1}^{445} {\text{x}}_{{\text{j}}} \times {\text{w}}_{{\text{hj }}} + {\text{w}}_{0 } } \right),\quad {\text{Or}}\;\;{\text{``w}}_{0 } {:}\;Bias{\text{''}} $$
  - Propagation from the hidden layer to the output layer using the formula (2):
    2 $$ y = f\left( {\mathop \sum \limits_{h = 1}^{445} z_{h} \times v_{h} + v_{{0{ }}} } \right),\quad {\text{Or}}\;\;{\text{``v}}_{0} {:}\;Bias{\text{''}} $$
    $\quad \quad \bullet f\left( x \right)$ is the activation function (sigmoid): $f\left( x \right) = \frac{1}{{\left( {1 + e^{ - x} } \right)}}$.
  - Calculation of the error between the desired output s and output y using formula (3):
    3 $$ \delta k = y\left( {1 - y} \right)\left( {s - y} \right) $$
  - Calculation of the error propagated on the hidden layer using the following formula (4):
    4 $$ \delta i_{h} = z_{h} \left( {1 - z_{h} } \right)\mathop \sum \limits_{k = 1}^{445} v_{h} \delta k{ } $$
  - Weight correction between the output layer and the layer hidden by formula (5):
    5 $$ \begin{aligned} \Delta v_{h} & = n \delta k z_{h} \\ \Delta v_{0} & = n \delta k \\ \end{aligned} $$
    $\quad \quad \bullet n$: rate of learning, with 0 < *n* < 1.
    Hence:
    $$ \left\{ {\begin{array}{*{20}l} {v_{h}^{{\left( {i + 1} \right)}} = v_{h}^{\left( i \right)} + \Delta v_{h} } \hfill \\ {v_{0}^{{\left( {i + 1} \right)}} = v_{0}^{\left( i \right)} + \Delta v_{0} } \hfill \\ \end{array} ,} \right.\quad \left( {\text{i}} \right){:}\;{\text{iteration}} $$
  - Correction of the connection weights between the hidden layer and the input layer by:
    6 $$ \begin{aligned} \Delta w_{hj} & = n \delta i x_{j} \\ \Delta w_{h0} & = n \delta i_{h} \\ \end{aligned} $$
    Hence:
    $$ \left\{ {\begin{array}{*{20}l} {w_{hj}^{{\left( {i + 1} \right)}} = w_{hj}^{\left( i \right)} + \Delta w_{hj} } \hfill \\ {w_{h0}^{{\left( {i + 1} \right)}} = w_{h0}^{\left( i \right)} + \Delta w_{h0} } \hfill \\ \end{array} ,} \right.\quad \left( {\text{i}} \right){:}\;{\text{iteration}} $$
  - Loop to step (c) as long as *δk* ≠ 0 or the number of iterations is $\le$ 10
  - Loop to the step (b) until browse all the instances of the learning base.
7. *Test and Results* By testing our algorithm with the available goodwares, we had the following results:
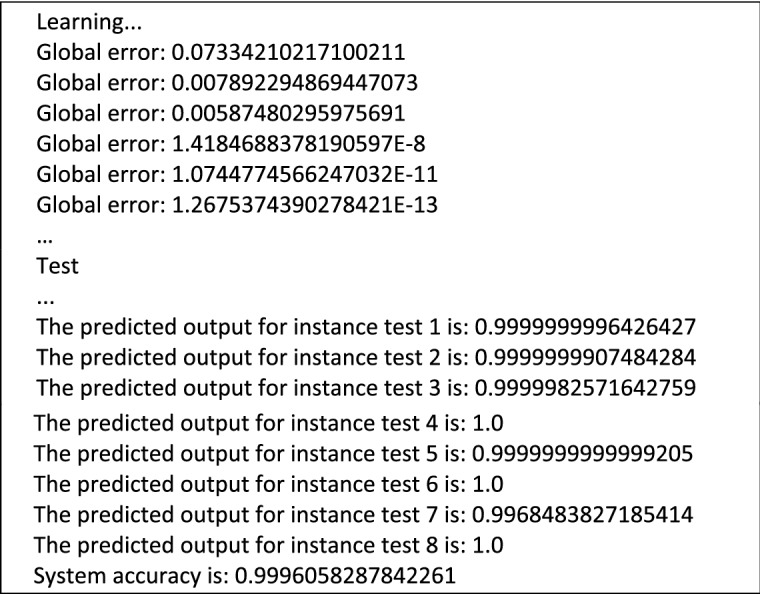
  In this phase, 30 instances (80%) were taken for learning and the rest (8 instances) for testing.
  It was found that the global error decreased during the learning phase, on the other hand 3 predicted outputs are equal to 1 and others are approximate to 1, which means that our model correctly classified the goodwares, with an accuracy of 99.96%.
8. *Learning and testing with Weka* In order to prove that the developed model works well, we used the Weka tool to form a multilayer perceptron. An ARFF file (a file format used by Weka to save the data) was constructed from the 38 instances that were generated previously.

**Figure 3:**
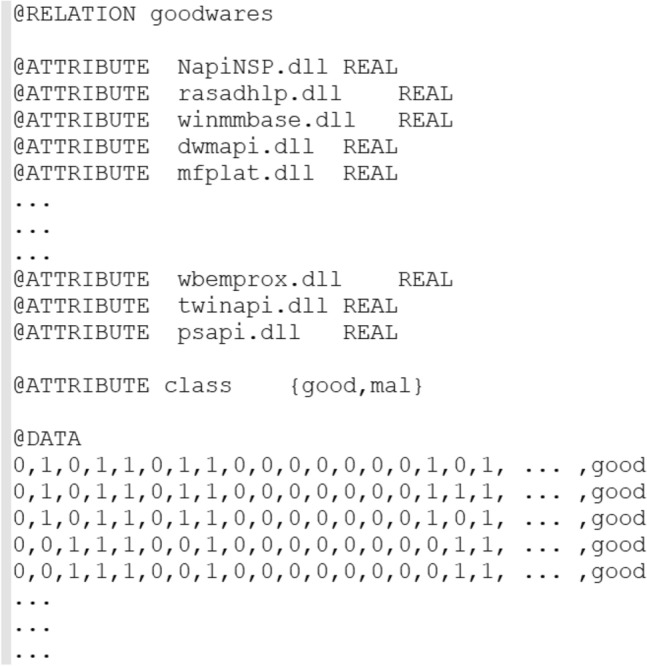
The dataset in ARFF format.

Using these data (Fig. 3) In Weka, we made the classification by Weka's multilayer perceptron function, with 80% of training data.

After the construction of the model, using Weka's tool, the model succeeded to classify correctly all instances of the test (20% of the dataset) with 100% accuracy, which promotes the proper functioning of our own model, and shows the power of artificial neural networks to solve software classification problems. However, these good results re-main incomplete because the dataset used contains only the elements labeled goodwares, which means that the learning base is not rich enough.

Thus, we sought to enrich our dataset, and we suggested some deep models for artificial neural network to solve the classification problem. The new implementation is made by Python language. For each proposed model, we followed the steps: prepare the data, then define, compile, adjust, and evaluate the model of network, and at the end make predictions.

- *Data preprocessing* or preparation of neural network model data, such as input and output values. We used the NumPy library to load the data in tabular form, which is ideal for our neural network in Keras. We divided our data set into two: a learning set and a test set. In addition, we used scikit-learn to train the model through the learning set, and test through the test set.
- *Definition of the network* The neural network models in Keras are defined as a sequence of layers, their container is the “Sequential” class to which one must create an instance, and add layers in the order in which they can be connected (Input, Hidden and Output Layer).
- *Compiling the network* After defining the network, we must specify some parameters such as the optimizer and loss function (The loss function is used to measure the error between the calculated result and the desired result). We also need to set the metric parameter on accuracy for our classification problem.
- *Adjusting the network* Adjusting the network means adapting the weights by doing the workout on a learning basis. Back-propagation requires the network to be trained for a specific number of iterations called epochs, each epoch is partitioned into batches, the batch size is the number of lines in our dataset entered into the network before the weights are updated in an epoch.
- *Evaluation of the network* Evaluating the network means estimating its performance on a test set. It provides an idea of how it works on new data.
- Predictions

## Results and discussion

### The proposed model “multilayer perceptron (MLP)” for the binary detection (goodware and malware (ransomware))

**Figure 4:**
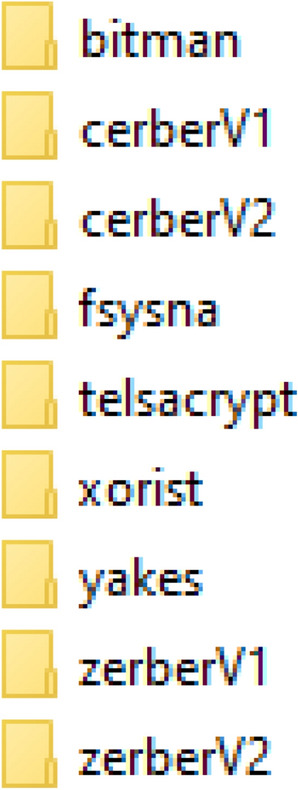
The classes of ransomwares.

**Figure 5:**
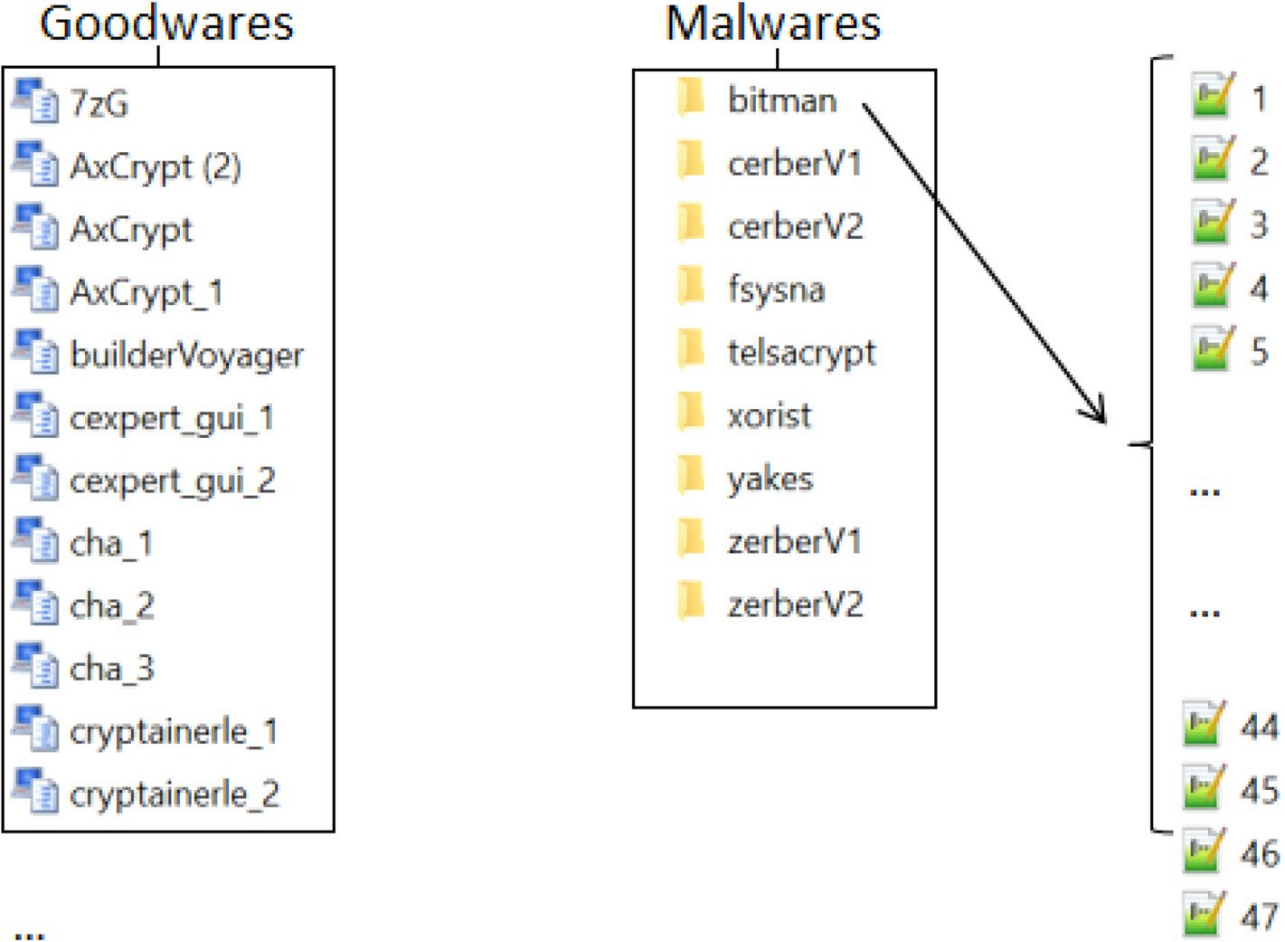
Dataset (goodware & malware).

To enrich our previous database, which contains 38, files representing goodwares (goodware class), we added 427 files representing ransomware (from our previous research^9^), the 427 files belong to 9 different ransomware families, as shown in the figure (Fig. 4):

We added these files to a second class: malware class. Hence, our database (Fig. 5) contains 465 different files.

**Figure 6:**
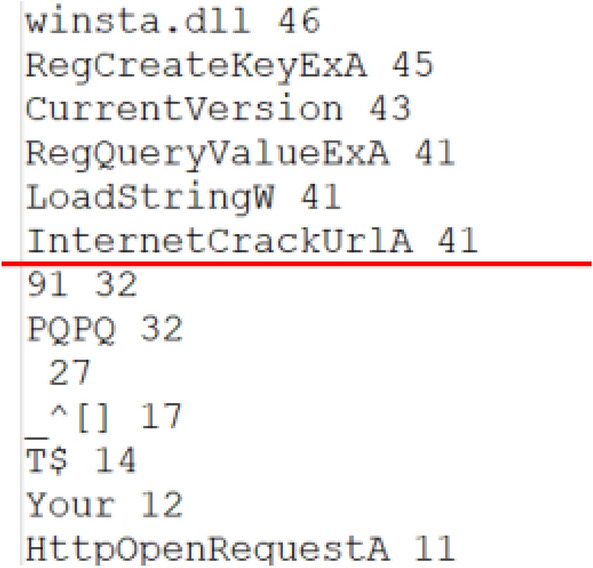
Elimination of irrelevant strings.

For each of the 9 ransomware families, we gathered all the strings contained in all the files of the same family (for example, for the Bitman class, we took all the strings that are in the 47 files), then we sorted them in descending order according to their number of occurrences, then we eliminated all the strings that have a number of occurrences below a certain threshold (Fig. 6). For the threshold, it was determined by eliminating all the chains that are found after a fall or an acute descent of the number of occurrences.

*For this example* We can stop at the threshold 41 because after this number there is a fall in the number of occurrences from 41 to 32 and then to 27, 17…etc. And we can also see the appearance of chains that do not make sense. (The same work is done for the goodware class) After that, we gathered all the results in the same file, and we extracted the distinct strings, the set of distinct strings represents our vector V1 of size 1410. Then, we instantiated all the files in our database by putting “1” if the vector string exists in the file, and “0” if not. Finally, we labeled the goodware class with “0”, and the malware class with “1”. Thus, we obtained 465 instances (lines). Each instance is composed of Boolean values that represent and characterize the associated file.

We have separated our database that contains 465 dataset into two sets: 80% of the data for learning (372 samples) and 20% for testing (93 samples). The architecture of our neural network was as follows:

- *The input layer* We have specified a fully connected layer with 1410 input variables.
- *The hidden layer* we chose to put a single hidden layer fully connected to 100 nodes and we used the ReLU activation feature.
- *The output layer* Fully connected layer, we put a single node using the sigmoid activation function to ensure that the only output on our network is either 0 for goodware or 1 for malware.

**Figure 7:**
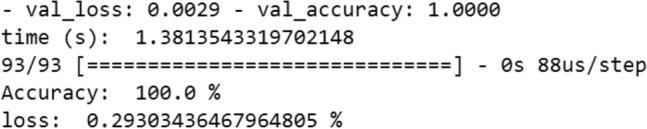
Evaluation of the MLP on the goodware and malware dataset.

To compile the model, we used cross entropy (binary_cross_entropy) as a loss function, and we used the optimizer Adam, which is a gradient descent algorithm that gives good results (Fig. 7).

The model was adjusted in 4 epochs with a lot size of 40.

Thus, our model gave an accuracy of 100%, which is perfect, in a training time of 1.38 s.

### Classification of nine families of ransomware

After the implementation of the multilayer perceptron, we had good results when we applied it to the binary classification (goodware or malware). We decided to test the same model to do the Malware classification, precisely the 9 families of ransomware (For the choice of the 9 classes, we based on the paper^22^ the authors created a platform called MoM that allows to make the dynamic analysis of Malwares and among the malwares used, there was “Ransomware”). Based on the previous work^9^, we took the vector V1 with the size 1089, with the extracted instances which were 437, we formed a multilayer perceptron on 80% of these data (349 samples), and tested it on the remaining data.

#### Classification with ANN (multilayer perceptron)

- *Model Definition* The model is defined in 3 layers (an input layer, a hidden layer and an output layer), knowing that the output layer has 9 neurons (corresponds to 9 ransomware categories) with the softmax activation function, for the purpose of making a classification of 9 classes.

We have compiled this model using the Adam optimizer and the multi-class logarithmic loss function (categorical).

**Figure 8:**
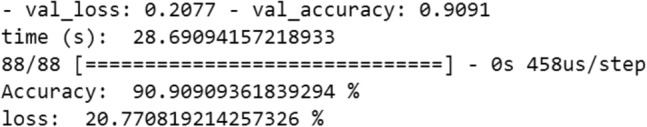
Evaluation of the MLP on the 9 classes of ransomware.

**Figure 9:**
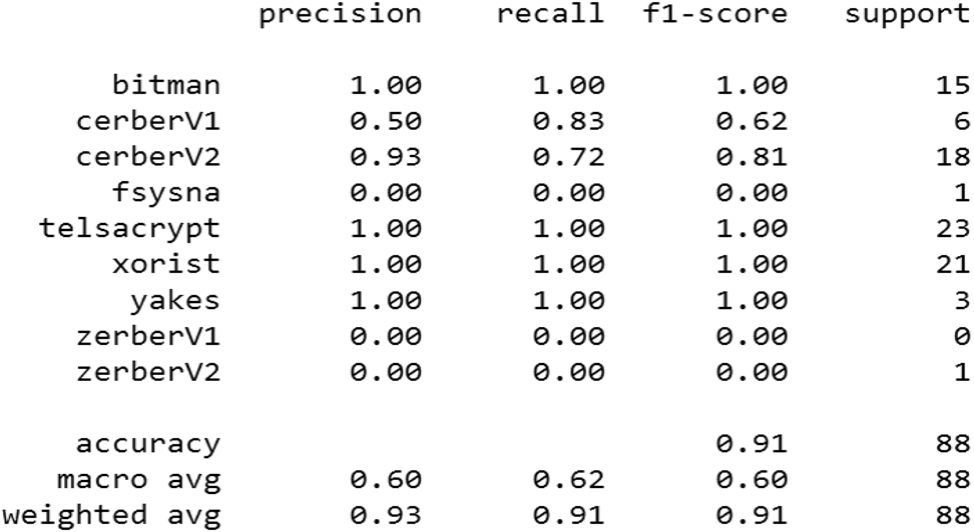
The metrics of the multilayer perceptron.

- *Evaluation* The model is evaluated in 3 epochs with a batch size of 5 lines, we got the following results (Fig. 8)
- Classification report (Fig. 9):

The accuracy of the model is 91%. If we compare with the results of the previous work^9^ we notice an increase in accuracy, from 70 to 91%. This is due to the good methodology followed and it also means that the developed model works well by perfectly classifying 4 classes without any error, except 2 classes (ZerberV1, ZerberV2) with the recall rate equal to 0.

#### Classification with CNN

- *Model Definition* The model is formed by two convolutional layers of 32 filters, each one followed by a grouping layer, then a flattening layer, and finally a layer fully connected to 9 neurons activated by the softmax function.

We have compiled this model by the Adam optimizer using the multi-class logarithmic loss function.

**Figure 10:**
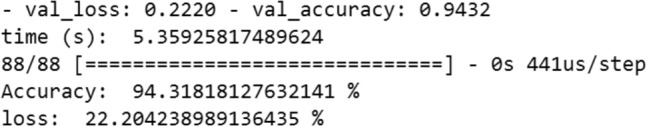
Evaluation of the CNN on the 9 classes of ransomware.

**Figure 11:**
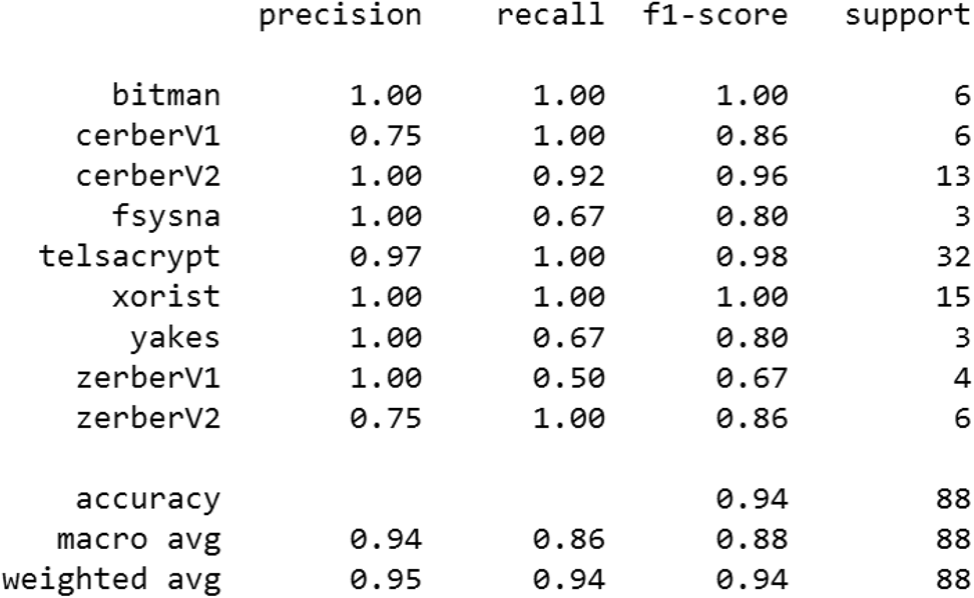
The metrics of the CNN.

- *Evaluation* The model is adjusted in 4 epochs with a batch size of 5 lines (Fig. 10)
- Classification report (Fig. 11):

The accuracy of the model is 94%; we can see that all accuracies are more than 0.75. So, the model has the ability not to label a negative sample as positive.

#### Classification with RNN

- *Model Definition* In this model, we used the Embedding layer in order to prepare the 427 available files, so that they would be acceptable by the recurrent model, then we used the LSTM (Long Short-Term Memory) recurrent layer, preceded by a Dropout layer and followed also by another Dropout, after we added the Flatten layer, and at the end the 9-neuron output layer activated by the softmax function.

We compiled this model by the Adam optimizer using the multi-class log loss function.

**Figure 12:**
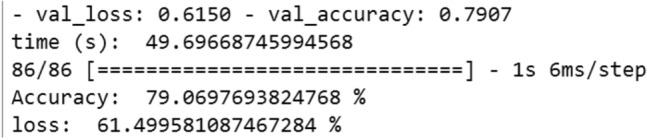
Evaluation of RNN.

**Figure 13:**
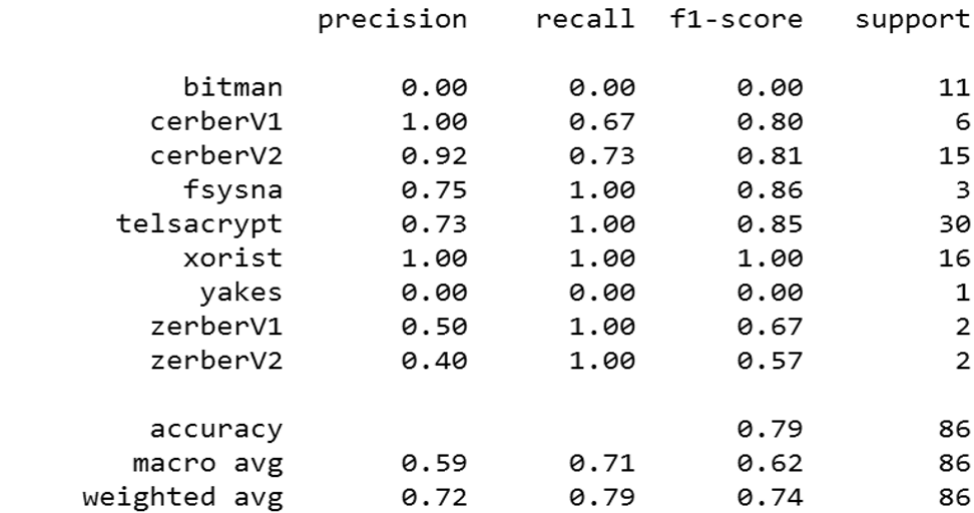
Metrics of RNN.

- *Evaluation* The model is adjusted in 3 epochs with a batch size of 20 lines (Fig. 12)
- Classification report (Fig. 13):

The accuracy of the model is 79% with a recall of the “Bitman” class is 0.

### Comparison between the different neural network

The Table 1. Shows a comparison of several classification methods between the different types of neural networks.

**Table 1:** Comparison of the classification between the different types of neural networks.

Type	Our results	Related works	Related works results
ANN	*Contribution 1*: The binary detection (malware and goodware) gave us an accuracy of **100%**	^7^	The accuracy for the classification of malware is 97.8%
	*Contribution 2*: The accuracy for classification (MLP) of nine families of ransomware is **91%**	^9^	The accuracy for the classification of ransomware: Using ANN is ≈ 70% Using BN is ≈ 49%
CNN	The accuracy for classification of nine families of ransomware is **94%**	^11^	They obtained in the results an accuracy of 98%
RNN	The accuracy for classification of nine families of ransomware is **79%**	^13^	No accuracy value
		^16^	ARI-LSTM (L = 5) = 0.93 (93%) ARI-LSTM (L = 8) = 0.91 (91%)

the values in bold are the results of accuracy obtained.

## Conclusion

This work is the continuation of the work^3^, we have implemented a neural network (MLP) to treat the binary classification (goodware/malware) as well as ransomware classification. In order to perform this classification, we used two sets of data: One for goodware and malware (binary classification) and the other for ransomware (used in the work^9^), we took 80% of this data set as a learning data and we kept the rest for the tests. The aim is to evaluate if the implemented model detects the instances correctly or not.

In the work^3^, the model was tested on binary classification and classification of 9 ransomware families using only artificial neural networks, in this work, an improvement was made by further testing the model on other types of neural networks, and the test was performed also on: CNN & RNN. The results obtained show that our model detects correctly and perfectly the instances using the binary classification with a rate of 100%, in the other hand; we found a rate of 91% using ANN, 94% using CNN and 79% using RNN. According the previous work^9^ based on ANN method, we realized an improvement, and the actual work was an occasion to perform other methods such us CNN and RNN. As limitations of the current work, we did not have a large dataset that allows us to improve learning and have a high recognition rate, because we based on a dataset that is not very sufficient. And as another limitation, in general, when we work with neural networks, we know that they give relevant results but it lacks visibility for programmers, like if we are working in a “black box”, which prevents us from understanding what's behind. Therefore, we are working to build another rich dataset rich to get the best possible results using different machine learning techniques and why not other techniques to do ransomware detection.
